# Paragangliome non secrétant de l'organe de Zuckerkandl

**DOI:** 10.11604/pamj.2015.20.130.5968

**Published:** 2015-02-16

**Authors:** Ayoub Halfya, Khalid Elmortaji, Mohamed Elmrini, Younes Houry, Said Osman, Karime Tounsi, Redouane Rabii, Rachid Aboutaib, Mohamed Dakir, Adil Debbagh, Fethi Meziane

**Affiliations:** 1 Service d'Urologie du CHU Ibn Rochd de Casablanca, Maroc

**Keywords:** Tumeur rétropéritonéale, paragangliome, organe de Zuckerkandl, retroperitoneal tumor, paraganglioma, Zuckerkandl organ

## Abstract

Le Phéochromocytome extra-surrénalien ou paragangliome est une tumeur rare. Il dérive de la crête neurale du système neuroendocrinien et il est souvent sécrétant. La plupart de ces tumeurs sont situées dans les ganglions sympathiques abdominaux, y compris l'organe de Zuckerkandl adjacent à la bifurcation de l'aorte abdominale. Les Formes non fonctionnelles sont rares: moins de 50 cas sont rapportés dans la littérature. Nous rapportons le cas d'un paragangliome non sécrétant de l'organe de Zuckerkandl opéré au sein de notre service d'urologie ainsi qu'une revue de la littérature afin de discuter les différents aspects diagnostiques et thérapeutiques de cette entité tumorale rare.

## Introduction

Le paragangliome ou phéochromocytome extra-surrénalien, est une tumeur d'origine neuroectodermique se développant aux dépens des tissus chromaffines; il représente représente 10% des cas, cependant l'atteinte de l'organe de Zuckerkandl est de loin la localisation ectopique exceptionnelle dans sa forme non sécrétante.

## Patient et observation

**Figure 1:**
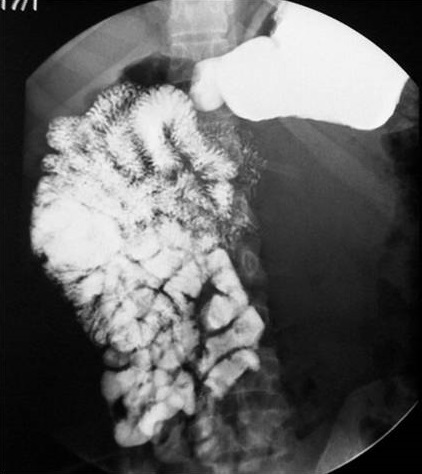
Transit du grêle: refoulement des anses intestinales de type extrinsèque

**Figure 2:**
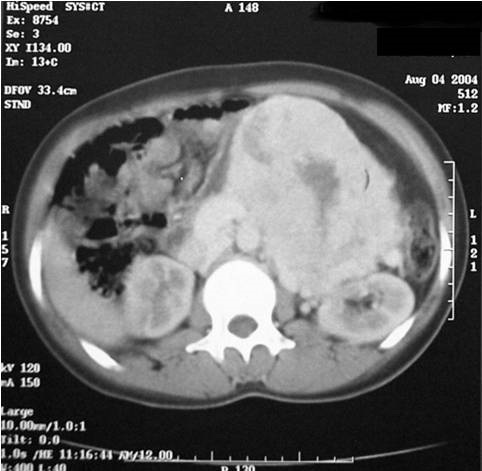
TDM abdominale avant et après injection de PDC: volumineuse masse tissulaire rétropéritonéale se rehaussant de façon hétérogène siège de zones de nécrose

**Figure 3:**
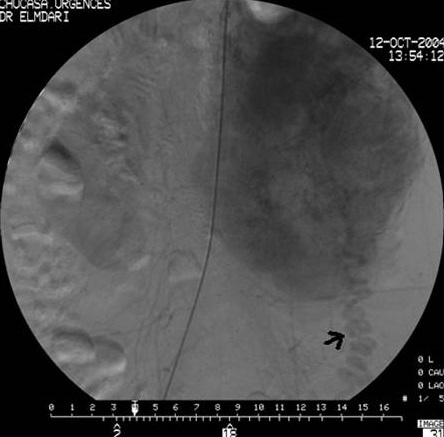
Artériographie: blush tumoral avec volumineuse veine de drainage (flèche)

**Figure 4:**
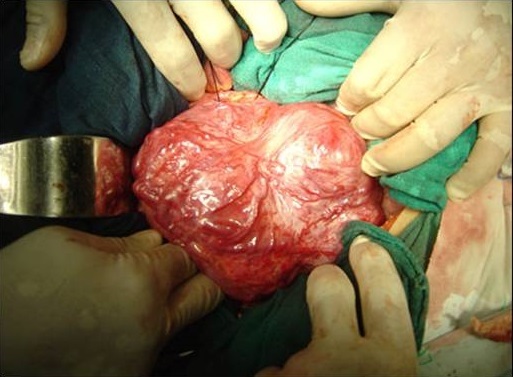
Aspect macroscopique de la pièce opératoire

N.D, jeune femme âgée de 20 ans, sans antécédents pathologiques particuliers, présente des douleurs de l'hypochondre et du flanc gauches depuis une année associées à des vomissements et des sueurs nocturnes sans fièvre ni d'hypertension artérielle ni de trouble de transit. L'examen clinique trouve une volumineuse masse au niveau du flanc gauche, ferme, mal limitée, fixe par rapport au plan profond sans autres signes cliniques associés. Une échographie abdominale montre une masse d’échostructure tissulaire hétérogène, bien limitée, d'allure intestinale. Un transit du grêle a été demandé montrant un refoulement des anses intestinales de type extrinsèque (Figure 1). Une Tomodensitométrie abdominale a révélé une masse tissulaire rétropéritonéale pré-rénale gauche mesurant 14 cm de grand axe, de structure hypodense hétérogène qui se rehausse après injection de produit de contraste avec persistance des zones hypodenses de nécrose (Figure 2). L'artériographie a montré un blush tumoral avec grosse veine de drainage (Figure 3). Le taux des dérivés méthoxylés urinaire est normal. La patiente fut opérée par un abord sous costal gauche et dont l'exploration a trouvé une tumeur rétro péritonéale de 14 cm de grand axe, en pré rénal gauche avec une importante néo vascularisation et sans adénopathie (Figure 4). L'exérèse tumorale est réalisée sans incidents, et les suites post opératoires étaient simples. L’étude anatomopathologique a confirmé le diagnostic de paragangliome non sécrétant de l'organe de Zuckerkandl.

## Discussion

Le paragangliome de l'organe de Zuckerkandl est une tumeur rare. Les manifestations cliniques de paragangliomes de l'organe de Zuckerkandl varient en fonction de son caractère sécrétant ou non sécrétant. Soixante-dix-sept pour cent de ces tumeurs sont sécrétantes, et seulement dans 23% des cas, le paragangliome de l'organe de Zukerkandl est non fonctionnel [1]. Les formes non fonctionnelles sont rares, seulement 50 cas qui ont été colligés dans la littérature [2–4]. Le plus souvent elles sont isolées, se caractérisant par leur aspect asymptomatique et des taux normaux des cathécolamines sanguins et urinaires. L’âge moyen de prédilection est de 40 ans avec des extrêmes allant 10 à 74 ans. Des observations exceptionnelles ont été rapportées chez le nouveau-né et au-delà de 80 ans. Le sex ratio est proche de 1. Le délai entre les premiers symptômes et le diagnostic est souvent long. Il s'agit le plus souvent d'une symptomatologie d'emprunt non spécifique à type de pesanteur abdominale, de signes urinaires, des lombalgies, et/ou une altération de l’état générale [5]. Lorsqu'ils sont secrétant, la pluparts des patients se présentent avec une hypertension paroxystique accompagnée de maux de tête, de sueurs, des palpitations et une rougeur du visage. Cependant, 10% des phéochromocytomes et paragangliomes sont de découverte fortuite [6], et dans 0,05% le diagnostic est porté en post-mortem [7]. L’échographie abdominale visualise la masse, affirme sa nature tissulaire et étudie son retentissement sur les organes de voisinage. Elle retrouve classiquement une masse échogène, homogène, bien limitée à contours nets, avec parfois des formations centrales d'allure kystique [8]. Néanmoins, sa sensibilité reste médiocre, elle ne détecte que les tumeurs de plus de 2cm de diamètre [2]. L′examen tomodensitométrique (TDM) a un intérêt majeur, Il affirme l′origine extra-surrénalienne et le caractère unique ou multiple de la lésion, ainsi que l′envahissement locorégional lymphonodal et l'effraction capsulaire. Il permet également de rechercher d’éventuelles métastases [9]. L′aspect tomodensitométrique d′un paragangliome est classiquement celui d′une masse solide, ronde ou ovale, homogène avec parfois un aspect kystique, nécrosée en son centre, ou calcifiée [10]. L′artériographie n′est pas spécifique. Au temps artériel, elle montre un aspect en rayon de miel, des vaisseaux anormaux tortueux et irréguliers. Au temps tardif, elle montre des shunts artério-veineux [11]. Quant à l′imagerie par résonance magnétique (IRM), elle décèle un signal faible intermédiaire en spin écho T1 et un hypersignal marqué en spin écho T2 se renforçant en deuxième écho, ce qui serait très évocateur avec une grande sensibilité [1, 6, 12].

En l′absence d′hypersécrétion de catécholamines, la scintigraphie à la méta-iodo-benzyl-guanidine (MIBG) n'est pas recommandée en préopératoire. En revanche, elle trouve une place prépondérante dans la surveillance post opératoire où elle permet la détection des récidives ou métastases. Elle a une sensibilité de 85% à 90% et une Spécificité de 95% [6]. La biopsie n′est pas fortement recommandée en raison du risque important d'une crise d′hypertension [1]. Le diagnostic de certitude est histologique. Sur le plan macroscopique, on est en présence d'une énorme tumeur arrondie, encapsulée, de consistance ferme et élastique, très vascularisée [13]. L′immunohistochimie permet d′affirmer le diagnostic de paragangliome grâce à l′identification plus précise de deux types de cellules, d′une part les cellules principales, disposées en nids “Zellballen” caractéristiques, marquées par la N.S.E. (neurone spécifique énolase), la métenképhaline, la chromogranine, la sérotonine, la gastrine, la V.I.P. (Vasoactive intestinal peptide), la somatostatine, la calcitonine, autres et d′autre part, les cellules sustentaculaires peu ou non visibles en microscope standard marquées par la protéine S100 et la G.F.A.P (protéine fibrillaire gliale acide) [14]. Il n′existe aucun critère histologique permettant de distinguer la bénignité de la malignité de la tumeur. Seule l′apparition de métastases dans un site où il n′existe pas habituellement de tissu paraganglionnaire ou la survenue d′une récidive locale ou à distance affirmeront la malignité [1, 12, 15, 16]. La chirurgie représente la base du traitement de ces tumeurs en raison de leur potentiel malin. L′exérèse, qui doit être totale pour être curative, nécessite parfois une extension aux organes adjacents. La possibilité de pratiquer cette chirurgie de manière radicale est estimée à 75% des cas [15]. Dans certains cas, une embolisation préopératoire a pu être proposée, permettant de réduire la vascularisation tumorale [17]. Comparée avec la chirurgie ouverte conventionnelle, la laparoscopie aboutit à une durée d′hospitalisation plus courte, une morbidité réduite et un rétablissement plus rapide. Par contre, on lui reprochait de pouvoir induire des modifications hémodynamiques susceptibles d′accentuer de façon dangereuse celles liées aux catécholamines libérées durant la période opératoire [18, 19]. Quoique des résections de large paragangliome allant jusqu’à 10 cm ont été rapportées dans la littérature avec succès [20].

## Conclusion

Le paragangliome rétropéritoneal non sécrétant est une tumeur très rare, parfois maligne, et souvent de découverte fortuite. L'exérèse chirurgicale demeure le traitement de choix, et seule l’étude anatomopathologique qui porte le diagnostic de certitude.
